# Isolated Pancreatic Tuberculosis in an Immunocompetent Host: A Diagnostic Challenge

**DOI:** 10.7759/cureus.94411

**Published:** 2025-10-12

**Authors:** Rawan AbouHatab, Isabel O'Shea, Brian Ho, Hassan Hatab, Anas Hatab

**Affiliations:** 1 Medicine, Lancashire Teaching Hospitals NHS Foundation Trust, Preston, GBR; 2 Internal Medicine, Royal Blackburn Teaching Hospital, Blackburn, GBR; 3 Gastroenterology, University of Liverpool, Liverpool, GBR; 4 Gastroenterology, East Lancashire Hospitals NHS Trust, Blackburn, GBR; 5 Gastroenterology, Royal Blackburn Teaching Hospital, Blackburn, GBR

**Keywords:** abdominal tb, endoscopic ultrasound, endoscopic ultrasound (eus), extra pulmonary tb, granulomatous inflammation, infection, pancreatic malignancy mimic, pancreatic mass, pancreatic tuberculosis

## Abstract

Pancreatic tuberculosis (TB) is rare and may closely mimic pancreatic malignancy, most often in immunocompromised patients. We describe the case of a man in his early 30s who presented with progressive epigastric pain, anorexia, fatigue, and 14 kg of weight loss. Computed tomography (CT) demonstrated a multicystic lesion in the pancreatic head encasing the coeliac axis with necrotic lymphadenopathy, appearances highly suspicious for malignancy. The case was reviewed at a regional multidisciplinary tumour meeting, and surgical resection was considered. Endoscopic ultrasound with fine needle biopsy initially demonstrated necrotising granulomatous inflammation; however, Ziehl-Neelsen staining was negative, and mycobacterial cultures were not requested. Six weeks later, a repeat biopsy demonstrated a culture positive for *Mycobacterium tuberculosis*. The patient was treated with a six-month course of anti-tuberculous therapy, with complete clinical and radiological recovery. This case highlights the importance of considering pancreatic TB even in immunocompetent patients and emphasises that mycobacterial cultures should be requested whenever granulomas are identified. Early repeat sampling can prevent misdiagnosis, unnecessary surgery, and delays in curative treatment.

## Introduction

Tuberculosis (TB) remains the leading cause of death from a single infectious agent, with an estimated 10.6 million new cases reported worldwide in 2022 [1]. Although TB most commonly affects the lungs, extra-pulmonary TB accounts for approximately 12.5% of cases. Of these, 11-16% involve the abdomen [1], where the gastrointestinal tract, lymphatic system, peritoneum, or abdominal viscera may be affected.

Pancreatic TB is among the rarest forms of abdominal TB. It typically presents as a cystic or solid lesion of the pancreas, with or without associated ascites or lymphadenopathy, and may closely mimic pancreatic malignancy both clinically and radiologically [2]. As there is no single diagnostic test that reliably confirms the condition, diagnosis usually depends on a combination of imaging, cytological or histological assessment, and microbiological confirmation. In selected cases, an empirical trial of anti-tuberculous therapy may also be considered [3].

Despite increasing recognition of extra-pulmonary disease, pancreatic TB remains under-recognised, particularly in immunocompetent patients. Most published cases describe relatively straightforward diagnostic pathways. We present an atypical case of isolated pancreatic TB in an immunocompetent young man, in whom diagnosis was delayed due to initial negative microbiological results. The infrequency of pancreatic TB may be partly attributed to the pancreas being an unfavourable environment for infection. Additionally, overlapping clinical and radiological features often lead to it being mistaken for pancreatic malignancy.

## Case presentation

A man in his early 30s was urgently referred to the pancreaticobiliary clinic from his general practitioner (GP) in August 2023 after reporting three months of epigastric pain, 14 kg of unintentional weight loss, fatigue, and anorexia. He denied respiratory, gastrointestinal, or systemic symptoms.

He had previously resided in Pakistan but had no known TB contacts, no personal history of TB, and no history of immunodeficiency. He was a non-smoker and did not consume alcohol.

On examination, he was afebrile with stable vital signs. There was no palpable lymphadenopathy or hepatosplenomegaly, and the abdomen was soft, non-tender, and without palpable masses.

Investigations

Initial blood tests, chest X-ray, and sputum microscopy with culture were unremarkable. Additional blood tests showed normal renal, hepatic, and haematological profiles, but inflammatory markers were raised with a C-reactive protein (CRP) of 37 mg/L and an erythrocyte sedimentation rate (ESR) of 32 mm/hour (Table 1).

**Table 1:** Blood markers on the initial testing (unit) CRP: C-reactive protein; ESR: erythrocyte sedimentation rate; ALT: alanine aminotransferase; ALP: alkaline phosphatase; GGT: gamma-glutamyl transferase; INR: international normalised ratio.

Blood markers on the initial testing (unit)	Result	Reference range
Haemoglobin (g/L)	132	130-180
White blood cell (x10^9^/L)	7.8	3.6-11.0
Platelets (x10^9^/L)	285	140-400
INR	1.4	0.8-1.2
Sodium (mmol/L)	138	133-146
Potassium (mmol/L)	4.2	3.5-5.3
Urea (mmol/L)	4.8	2.5-7.8
Creatinine (µmol/L)	67	59-104
Bilirubin (µmol/L)	16	<21
ALT (IU/L)	18	<41
ALP (IU/L)	116	30-130
GGT (IU/L)	45	<60
Albumin (g/L)	42	35-50
Adjusted calcium (mmol/L)	2.38	2.2-2.6
TIBC (µmol/L)	52	45-72
Ferritin (µg/L)	59	30-400
Amylase (IU/L)	69	30-118
CRP (mg/L)	37	<5
ESR (mm/hour)	32	0-13

**Figure 1:**
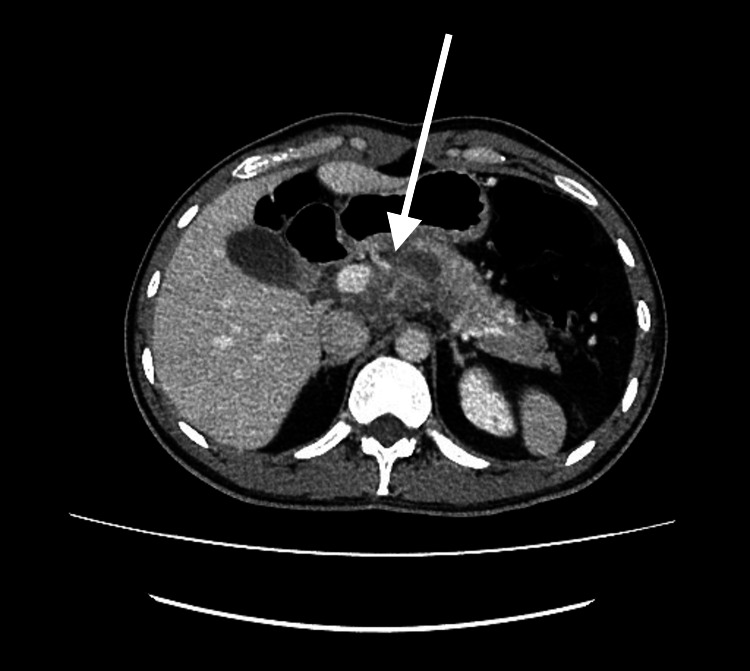
CT thorax abdomen and pelvis with contrast Contrast-enhanced CT showing a multicystic lesion in the pancreatic head/neck region encasing the coeliac axis, with necrotic lymphadenopathy.

A contrast-enhanced computed tomography (CT) scan of the thorax, abdomen, and pelvis revealed a multi-cystic lesion in the head and neck of the pancreas encasing the coeliac axis, with necrotic lymphadenopathy, raising suspicion for pancreatic cancer (Figure 1, arrows).

The patient was referred to the Pancreatic Rapid Diagnostic Service. Tumour markers were within normal ranges: carcinoembryonic antigen (CEA) 1.4 µg/L (reference range: 0-35 kU/L); carbohydrate antigen 19-9 (CA19-9) 28 kU/L (reference range: <37 U/mL). Screening for human immunodeficiency virus (HIV), hepatitis B, and hepatitis C was negative (Table 2).

**Table 2:** Infectious and tumour marker screening HIV: Human immunodeficiency virus; CEA: carcinoembryonic antigen; CA19-9: carbohydrate antigen 19-9.

Blood marker	Result
Hepatitis B surface antigen	Not detected
Hepatitis C antibody	Not detected
HIV antibody/p24 antigen	Not detected
CEA	1.4 (µg/L)
CA19-9	28 (kU/L)

Endoscopic Ultrasound

**Figure 2:**
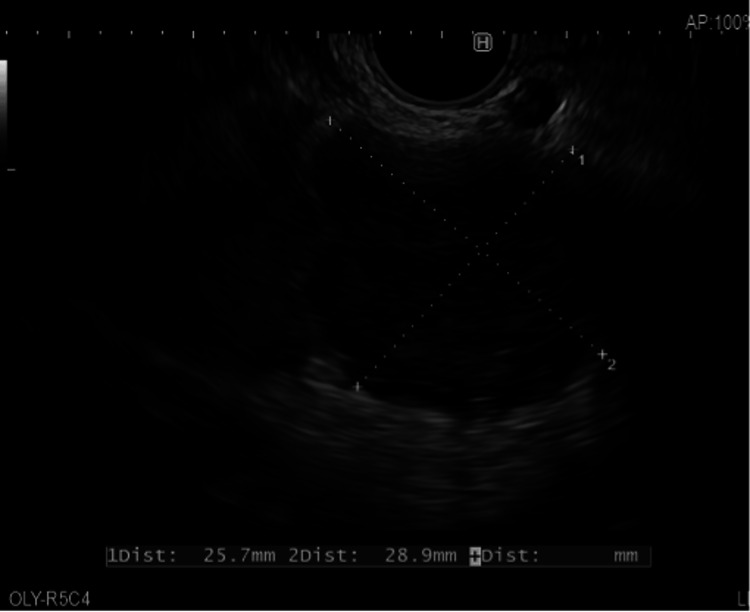
EUS-guided fine needle biopsy Fine needle biopsy of the pancreatic head lesion showing necrotising granulomatous inflammation. Granulomatous inflammation is indicated by the cross (×). Ziehl-Neelsen staining was negative. EUS: Endoscopic ultrasound

Endoscopic ultrasound (EUS) revealed a large mass with cystic and solid components in the pancreatic head that distorted the upper gastrointestinal anatomy, limiting access. Fine needle biopsy (FNB) sampled a solid material, which showed necrotising granulomatous inflammation without malignant cells. Ziehl-Neelsen (ZN) staining was negative (Figure 2).

**Figure 3:**
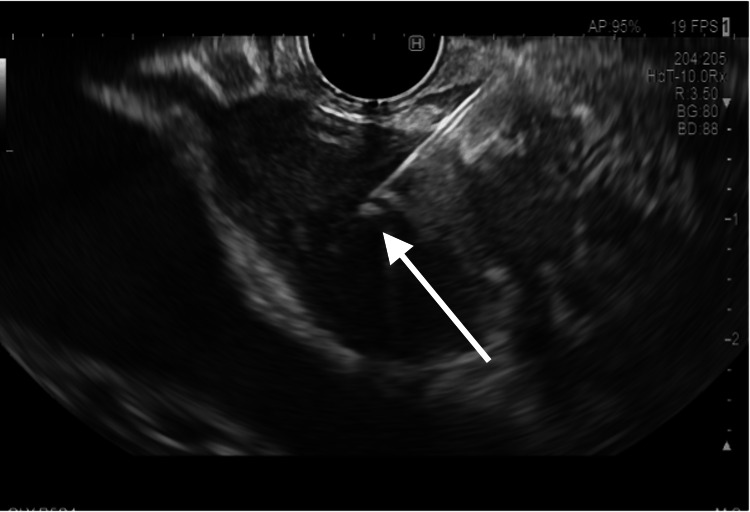
Hypoechoic lesion in the pancreatic head on endoscopic ultrasound (EUS) EUS image demonstrating a hypoechoic lesion in the head of the pancreas, without a visible cystic component. Fine needle aspiration was performed for microbiological analysis. Arrows indicate the hypoechoic lesion.

Given the inconclusive findings, a repeat EUS was performed. This demonstrated a hypoechoic lesion in the pancreatic head without cystic components. Fine needle aspiration (FNA) was successfully performed (Figure 3).

Microbiological Confirmation

FNA samples were obtained during the second EUS procedure and sent for mycobacterial culture. Although acid-fast bacillus staining was negative, the culture confirmed the presence of *Mycobacterium tuberculosis*.

Treatment

The patient was treated according to World Health Organization (WHO) guidelines for abdominal TB, with isoniazid, rifampicin, ethambutol, and pyrazinamide for an intensive two-month phase, followed by a four-month continuation phase of isoniazid and rifampicin [3].

Outcome and follow-up

At two months, he reported resolution of abdominal pain, anorexia, and weight loss. He developed mild pruritus during therapy, managed with chlorphenamine. He completed six months of treatment, returned to normal daily activities, and regained 10 kg in weight. Follow-up CT demonstrated regression of pancreatic and nodal abnormalities.

## Discussion

Pancreatic TB is an exceptionally uncommon manifestation of extra-pulmonary TB, even in regions where the disease is endemic, and it often mimics malignancy both radiologically and clinically [4]. Most published cases involve older or immunocompromised patients, particularly those with risk factors such as prior TB exposure, low socioeconomic status, or HIV infection [5]. In a systematic review of 166 cases, 25.3% of patients were HIV positive [6]. A variety of published case reports support this association [7-9]. Our case is unique because the patient was young, immunocompetent, and presented with isolated pancreatic involvement.

The rarity of pancreatic TB may be partly attributable to the pancreas being an unfavourable site for *Mycobacterium tuberculosis*, which may be explained by the anti-mycobacterial effect of pancreatic enzymes such as lipase [4]. Despite this, cases in immunocompetent individuals are becoming increasingly recognised [7,9].

Diagnosis remains a difficulty. Symptoms such as abdominal pain, weight loss and fatigue are non-specific, and may overlap with several other conditions, including malignancy. Alongside this, inflammatory markers were elevated (CRP 37 mg/L, ESR 32 mm/hour). This indicates an underlying chronic inflammatory state. Although these findings are consistent with extrapulmonary TB, they are also seen in pancreatic malignancy, which complicates the diagnostic interpretation.

Radiological findings added to the diagnostic uncertainty. CT revealed a cystic lesion with necrotic lymphadenopathy, which is indistinguishable from pancreatic malignancy [10,11]. The overlap further reinstates the need for early tissue diagnosis.

EUS with FNA plays a critical role in diagnosis. It enables differentiation between neoplastic, inflammatory, and infectious processes [12,13]. Our patient's EUS-FNB revealed necrotising granulomatous inflammation, which is typical of granulomatous diseases, such as sarcoidosis and TB. However, ZN staining was negative, and unfortunately, mycobacterial cultures were not requested at the time, representing a missed diagnostic opportunity. 

Reliance on ZN staining alone is insufficient to diagnose pancreatic TB. In extrapulmonary disease, bacilli are often sparsely distributed, which limits the sensitivity of staining. Therefore, when a granulomatous lesion is identified, mycobacterial culture and, where available, nucleic acid amplification testing (NAAT) should be performed as part of the initial diagnostic workup [6]. In our case, a correct diagnosis was only reached after repeated EUS-FNA, which showed a culture-positive result for *Mycobacterium tuberculosis*.

Currently, there are no formal guidelines or consensus diagnostic criteria for pancreatic TB, resulting in variations in clinical practice [6]. Owing to its rarity, clinical awareness remains limited, which can lead to misdiagnosis, invasive investigations and delayed treatments. Ray et al. reported a series in which 6 of 16 patients underwent surgery before TB was correctly identified [14]. Similar cases underscore the importance of heightened clinical awareness to prevent unnecessary surgical intervention [15-17]. Siddeek et al. reported a similar case in which pancreatic TB mimicked malignancy before the diagnosis was finally confirmed [17]. Crucially, granulomatous inflammation should always prompt mycobacterial evaluation [18]. In our case, neither mycobacterial culture nor NAAT was performed at the initial biopsy, representing a missed opportunity. The correct diagnosis was only made after a repeat EUS-FNA provided a culture-positive sample.

Our patient responded well to a six-month course of standard therapy, consistent with WHO guidance [3]. He showed clinical improvement (reporting resolution of pain and weight gain), biochemical normalisation (of inflammatory markers), and radiological regression (of pancreatic and nodal lesions on follow-up CT).

This case reinforces three critical lessons. Firstly, mycobacterial testing should be performed in all patients with granulomatous inflammation. Secondly, EUS-FNA testing is crucial in diagnosis, especially when the initial results are indeterminate. Finally, clinicians should maintain a high index of suspicion for pancreatic TB in patients from endemic regions, even if there are no evident predisposing risk factors. 

## Conclusions

Pancreatic TB is a rare but important differential diagnosis for pancreatic lesions, particularly in patients from TB-endemic regions. Because its presentation often mimics pancreatic malignancy, diagnosis relies on EUS-FNA and subsequent microbiological evaluation. This case demonstrates that granulomatous inflammation on biopsy should warrant mycobacterial studies and that repeating EUS-FNA may be decisive in reaching the correct diagnosis. Early recognition and timely initiation of anti-tuberculous therapy can lead to excellent patient outcomes and help avoid unnecessary invasive procedures.

However, this is only a single-patient case report, meaning there is limited follow-up. Further studies and long-term outcomes are needed to better understand diagnostic approaches and prognosis in similar presentations.
